# Unlocking the genetic potential of *Triticum urartu* for wheat improvement: a review

**DOI:** 10.3389/fpls.2026.1894970

**Published:** 2026-07-17

**Authors:** Veronica F. Guwela, Martin R. Broadley, Malcolm J. Hawkesford, Surbhi Grewal, Moses F.A. Maliro, James Bokosi, Workie Zegeye, Julie King

**Affiliations:** 1 International Maize and Wheat Improvement Centre (CIMMYT), Texcoco, Mexico; 2 Rothamsted Research, West Common, Harpenden, United Kingdom; 3 School of Biosciences, University of Nottingham, Loughborough, United Kingdom; 4 Department of Crop and Soil Sciences, Lilongwe University of Agriculture and Natural Resources, Lilongwe, Malawi; 5 John Innes Centre, Norwich, United Kingdom

**Keywords:** wheat, Triticum urartu, accessions, agronomic traits, introgression, molecular markers, cytogenetics, doubled haploids

## Abstract

The narrow genetic base of cultivated wheat (*Triticum aestivum* L.) remains a major constraint to genetic improvement, particularly in addressing current and emerging production challenges. Expanding this genetic base is essential to overcome yield plateaus and meet the food demands of a growing global population. Wild relatives and ancestral progenitors of wheat harbour extensive, underutilized genetic diversity that can be harnessed for crop improvement. Among these, *Triticum urartu*, the A-genome donor of bread and durum wheat, presents a potentially valuable reservoir of traits related to biotic and abiotic stress tolerance, and grain quality. In this review, we summarise current knowledge on the potential of *T. urartu* as a source of resistance to major wheat diseases including powdery mildew, stem rust (notably the highly virulent race Ug99), leaf rust, and stripe rust. In addition, we discuss the potential of *T. urartu* as a source of drought and heat tolerance, enhanced photosynthetic traits and quality associated traits. We further highlight the successful introgression of *T. urartu* chromosomes into diploid, tetraploid, and hexaploid wheat backgrounds, demonstrating its compatibility across multiple ploidy levels. Advances in doubled haploid (DH) technology have recently accelerated the generation of homozygous wheat–*T. urartu* introgression lines, thereby facilitating rapid trait fixation and efficient germplasm development. The application of molecular marker technologies, including SNP-based kompetitive allele-specific PCR (KASP) assays, has further improved the characterization of *T. urartu* genetic diversity and the precise tracking of introgression lines. The increasing availability of validated SNP datasets in public repositories and the development of scalable high- and -medium throughput genotyping platforms are further accelerating wheat–*T. urartu* introgression programs.

## 1 Introduction

Grown on an estimated 218 million hectares of land with about ~780 million tonnes of grain harvested annually (Shahbandeh, 2025), hexaploid bread wheat (*Triticum aestivum ssp vulgare*) and tetraploid durum (*Triticum turgidum ssp durum*) are the two main species of wheat that are widely cultivated (Dvořák et al., 1993). Hexaploid wheat consists of three sub-genomes: AA, BB, and DD from diploid *T. urartu* (A^u^A^u^), an unidentified species related to *Aegilops speltoides* (SS) and diploid *Aegilops tauschii* (DD), respectively (Dubcovsky and Dvořák, 2007; Dvořák et al., 1993; Feldman and Levy, 2015). Domestication and polyploidy speciation resulted in loss of genetic diversity of cultivated wheat compared to progenitor species and wild relatives (Dubcovsky and Dvořák, 2007; He et al., 2019). Limited variation and full exploitation of cultivated wheat for breeding over the years, has also posed a challenge to further wheat improvement (Feldman and Sears, 1981), and increased vulnerability of wheat to different biotic and abiotic stresses (Dubcovsky and Dvořák, 2007; Feldman and Sears, 1981). In Sub- Saharan Africa for instance, wheat diseases such as yellow rust and stem rust have caused yield losses of up to 100% and lead to the collapse of dominant wheat varieties (Tadesse et al., 2018). Across the globe, an estimated 90% of wheat varieties are reported susceptible to wheat stem rust as new and more virulent races are emerging (Figueroa et al., 2018; Singh et al., 2011). This, coupled with the breakdown of major disease resistance genes for some important wheat diseases, poses a threat to global food security. *T. urartu*, though not widely exploited, shows potential for several agronomically important traits that could be useful for enriching the genetic base of cultivated wheat. Advances in plant genetics and genomics have accelerated wheat-progenitor species/wild relatives pre-breeding programs and allowed the transfer of alien genomes into cultivated wheat. Here, progress made in the identification and utilisation of *T. urartu* agronomic and quality traits is summarised. In addition, techniques for characterising the introgression lines are discussed.

## 2 General description Of *Triticum urartu*

**Figure 1:**
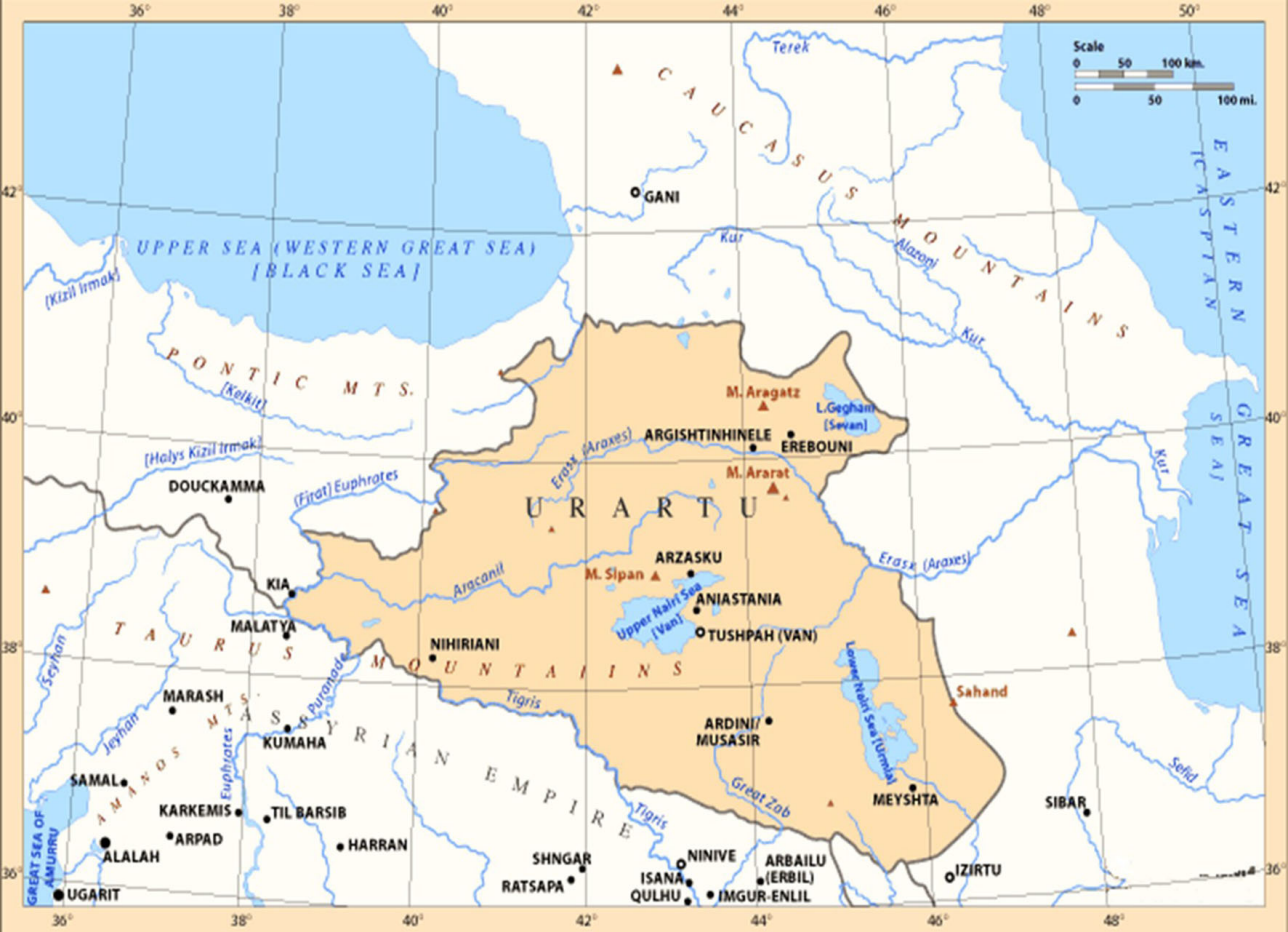
Geographic map of the fertile crescent highlighting the regions identified as the origin of *T. urartu*. Source: World history encyclopaedia.

*Triticum urartu* Thum. ex Gandil. (2n = 2x = 14; genome A^u^A^u^), also known as red wild einkorn wheat, is an annual wild diploid species belonging to the Poaceae family. *T. urartu* is native to the Fertile Crescent (**Figure 1**), including countries such as Turkey, Lebanon, Iraq, and Iran (Johnson, 1975; Xiao et al., 2018). It is recognized as one of the most significant wild relatives of cultivated wheat, having contributed the A genome of both modern hexaploid and tetraploid wheat. An AFLP marker-based analysis of 200 *T. urartu* accessions collected across the Fertile Crescent (**Figure 1**) revealed that the species’ centre of genetic diversity is in the northernmost part of the region, particularly around the Syrian Turkish border (Baum and Bailey, 2013).

**Figure 2:**
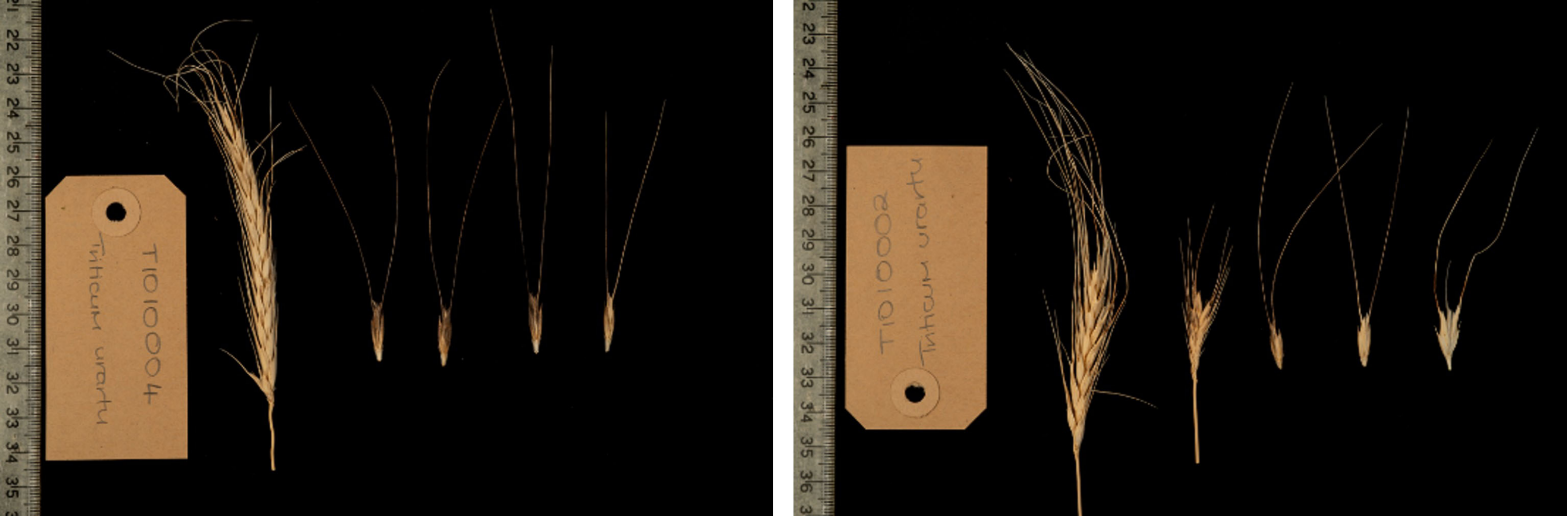
Spikelets and ears morphology of two *T. urartu* accessions maintained at the genetic resource unit (GRU) at John Innes Centre Photo credit: Phil Robinson.

Initial studies of *T. urartu* suggested that it is the B genome donor of cultivated wheat (Johnson, 1975), however, through observing the pairing behaviour of marked A and B telocentric chromosomes of 14 *T. aestivum-T. urartu* hybrids at meiosis, it was discovered that *T. urartu* is the A genome donor of *T. aestivum* (Chapman et al., 1976; Dvořák, 1976). This finding was further supported by Dvořák et al. (1993) who analysed polymorphisms of repeated nucleotide sequence profiles of the A genomes and confirmed that *T. urartu* contributed to the A genomes of *T. turgidum*, *T. timopheevii*, and *T. aestivum*. Similary, *Triticum monococcum* (2n = 2x =14, A^m^A^m^) had previously been regarded as the A−genome progenitor of hexaploid wheat (Sax, 1922; Lilienfield, 1951; Moghaddam et al., 2000; Baum and Bailey, 2004), however, subsequent cytogenetic and molecular evidence demonstrated that *T. urartu* rather than *T. monococcum* is the direct donor of the A genome of hexaploid and tetraploid wheat. Phylogenetic analysis of some *T. urartu* accessions also provide molecular evidence that *T. urartu* is the A-genome donor of hexaploid wheat (Adhikari et al., 2022; Luo et al., 2015), and that the species that was involved in the first wheat allopolyploidization was very similar to *T. urartu* but had diverged from it circa 1.3 MYA (Levy and Feldman, 2022; Marcussen et al., 2014; Li et al., 2022). Genome-wide sequencing of millions of high-quality SNPs from representative accessions of *T. urartu* collected around the world showed that the direct donor of the wheat A sub-genome originated from northwestern Syria (Wang et al., 2022). High quality and draft genome assemblies have revealed that the genome size of *T. urartu* is approximately 4.94 Gb (Akhunov et al., 2005; Ling et al., 2018, Ling et al., 2013). Morphologically, *T. urartu* has small anthers (3 per floret), a prominent second tooth on the sterile glume, a two awned spikelet (**Figure 2**), a pubescent leaf and reddish kernels (Kimber and Feldman, 1987; Castagna et al., 1997).

## 3 Important traits identified in *T. urartu* accessions

### 3.1 Disease resistant traits

Disease resistance is a major target trait for any breeding program, and it is an important trait for sustainability of yield in cultivated crops. Effects of diseases on wheat crop yields and quality have been widely studied (Laidig et al., 2021; Wellings, 2011). A number of wild relatives and progenitor species have contributed to sources of resistance for some diseases and subsequently contributed to food security (Friebe et al., 1996). Several wild relatives of wheat across the primary, secondary, and tertiary gene pools have been identified as valuable sources of disease resistance. In the secondary gene pool for example, *Aegilops speltoides* and Aegilops muticum have shown resistance to powdery mildew (Petersen et al., 2015; Liu et al., 2015) and Septoria nodorum blotch (Zhang et al., 2019) while *Aegilops searsii* has shown resistance to powdery mildew (Liu et al., 2011; Zhao et al., 2024). Similarly, *Triticum timopheevi* showed resistance to fusarium head blight (Steed et al., 2022). In the tertiary gene pool *Thinopyrum elongatum* species were identified as a source stem rust resistance (Yu et al., 2023), while *Thinopyrum intermedium* showed resistance to wheat streak mosaic virus (Ali et al., 2016). Several screening studies for *T. urartu* have also revealed some excellent disease resistance traits (**Table 1**) that could be useful for breeding for resistance to major diseases that are responsible for substantial crop losses in cultivated wheat.

**Table 1:** Summary of agronomic, quality, and stress tolerance traits identified in *T. urartu* and associated genes.

Trait type	Associated novel genes/key findings	Key references
Powdery mildew resistance	*Pm60* locus with *NB-LRR* genes; alleles *Pm60*, *Pm60a* and *Pm60b*	Zhao et al. (2020); He et al. (2018); Zou et al., (2018, Zou et al., 2022a); Liu et al. (2015); Zhang et al., (2016, Zhang et al., 2018)
Stem rust resistance	Resistance to races TTKSK (Ug99), QFCSC, and MCCFC	Rouse and Jin (2011)
Leaf rust resistance	Resistance identified at juvenile and adult stages	Tyryshkin et al. (2022)
Stripe rust resistance	Resistance to races CYR33 and CYR32; novel *YrU1* gene; *TuNAC69* transcription factor	Dhaliwal and Gill (1982); Ma et al. (1997); Xiao et al. (2018); Wang et al. (2020); Xu et al. (2022); Zou et al. (2022b)
Root lesion resistance	Resistance identified in five accessions	Sheedy et al. (2012); Mokrini et al. (2019)
Endosperm storage proteins	Glutenins (HMW-GSs, LMW-GSs) and gliadins	Bai et al. (2004); Caballero et al. (2009); Hu et al. (2010); Jiang et al. (2009); Luo et al. (2015); Zhang et al. (2015); Cuesta et al., (2015, Cuesta et al., 2017); Talini et al. (2020); Ahmadi et al. (2018)
Micronutrients	High Zn and Fe concentrations	Ma et al. (1997); Guwela et al., (2024a, Guwela et al., 2024b)
Starch synthesis	Novel waxy genes (*Wx-Au1b*, *Wx-Au1c*, *Wx-Au1d* and *Wx-Au1e*); *TubZIP28* transcription factor	Yan and Bhave (2000); Guzman and Alvarez (2012); Ortega et al. (2014); Zi et al. (2018); Song et al. (2020)
Photosynthesis	Enhanced flag-leaf photosynthesis and high stomatal conductance	Austin et al., (1982, Austin et al., 1986); Wang et al. (2008); McAusland et al. (2020)
Drought tolerance	High relative water content; high drought stress tolerance index; DREB genes	Kamal et al. (2010); Fricano et al. (2014); Pour-Aboughadareh et al. (2017); Aberkane et al. (2020); Rustamova et al. (2022)
Heat and cold tolerance	Novel polyamine oxidase (PAO) genes	Huang et al. (2006); Gao et al. (2017); Zhang et al. (2016); Gholizadeh and Mirzaghaderi (2020); Riaz et al. (2025)
Pre-harvest sprouting	*TaPHS1* allele associated with stronger seed dormancy	Liu et al. (2015)
Salinity tolerance	Novel *TuPr-1-rk* gene; novel Vesicle-Inducing Protein in Plastids genes (*TuVipp1* and *TuVipp2*)	Lu et al. (2018)

#### 3.1.1 Wheat powdery mildew *resistance in T. urartu*

Powdery mildew is a fungal foliar disease responsible for yield losses of up to 30% (Griffey et al., 1993; Zhao et al., 2020). A more economic and effective way of controlling powdery mildew is through the use of powdery mildew (*Pm*) resistance genes (He et al., 2018; Zhao et al., 2020). *T. urartu*, represents an important source of powdery mildew resistance mediated through nucleotide-binding leucine-rich repeat (NB-LRR) immune receptors that trigger hypersensitive response (HR) type of resistance associated programmed cell death (Zou et al., 2018). Identified on a dominant resistance locus (*Pm60*), in *T. urartu* accessions, the loci became the first to be characterised in *T. urartu* (Zou et al., 2018*).* Subsequent characterization of the allelic variants of *Pm60*, *Pm60a*, and *Pm60b* demonstrated further disease resistance and both functional conservation and allelic diversification within the locus (Zou et al., 2022a). The three allelic variants identified through these studies conferred resistance to wheat powdery mildew (*Blumeria graminis f.* sp. *Tritici*) race BgtE09, although reduced resistance spectrum was observed in *Pm60a* against other powdery mildew isolates, and this was likely associated with deletion of the LRR motif on *Pm60a*. Interestingly, Zhao et al. (2020) identified a non-functional *Pm60a* like allele (Pm60a″) in susceptible *T. urartu* accessions. Despite sharing 98.52% sequence identity with *Pm60a*, the presence of 58 single nucleotide polymorphisms (SNPs) and a three-nucleotide deletion likely contributed to the loss of the resistance function. Genome-wide studies in *T. urartu* have shown the functional significance of the NBS containing resistance genes involved in powdery mildew defense. Using the whole-genome sequence of *T. urartu*, Liu et al. (2017) identified 461 full-length proteins containing four classes of NBS resistance domains, including NBS-LRR proteins, distributed across all seven chromosomes, with chromosome 7A harbouring the highest number of resistance related sequences. Expression profiling demonstrated that six NBS genes were differentially expressed in response to *B. graminis* infection at the two-leaf stage, suggesting their potential involvement in early defense responses. In addition, characterization of resistance gene analogues (RGAs) on chromosome 7A^u^L of *T. urartu* revealed 126 RGAs, including 30 localized within the powdery mildew resistance gene (*PmU*) region, of which 14 possessed corresponding expression data in the *T. urartu* transcriptome database. Expression analysis of the 14 *PmU*-RGAs and *Pm60* after inoculation with *Bgt* race *E09*, showed that *Pm60* was specifically expressed in the *T. urartu* accession carrying *PmU*, but not in susceptible accessions (Zhang et al., 2018). Furthermore, immune (IM) and hypersensitive reaction (HR) responses studies in *T. urartu* identified additional candidate immune receptor genes positively associated with powdery mildew resistance in *T. urartu* accessions (Zhang et al., 2016, Zhang et al., 2018).

#### 3.1.2 Wheat stem, leaf and stripe rusts

Wheat rusts are one of the major wheat production constraints globally. Together, wheat stem rust (*Puccinia graminis f.* sp. *tritici*), leaf rust (*Puccinia triticina*) and stripe rust (*Puccinia striformis f.sp. tritici*) have caused substantial crop losses and led to the collapse of dominant varieties (Tadesse et al., 2018). *Triticum urartu* has also been identified as an important source of resistance against major wheat rust diseases, particularly stem rust caused by *Puccinia graminis f.* sp. *Tritici*. Screening of 205 *T. urartu* accessions revealed that approximately 95% exhibited resistance to the highly virulent stem rust race TTKSK (Ug99) (Rouse and Jin, 2011), a race of global concern due to its virulence against numerous resistance genes widely deployed in modern wheat cultivars (Singh et al., 2015). The same study further identified resistance to additional stem rust races, including QFCSC and MCCFC, demonstrating the broad-spectrum resistance potential present within *T. urartu* germplasm. Beyond stem rust, resistance to leaf rust caused by *Puccinia triticina* was also detected in three *T. urartu* accessions evaluated at both juvenile and adult plant growth stages (Tyryshkin et al., 2022).

Resistance to stripe rust was first reported in *T. urartu* in the early 80s (Dhaliwal and Gill, 1982). More recently, resistance to two major Chinese stripe rust races CYR33 and CYR32 was evaluated in 147 *T. urartu* accessions. The study revealed a higher frequency of resistant to CYR33 compared to CYR32, with several accessions exhibiting resistance to both races. Among the resistant accessions, few exhibited high resistance, while a majority were moderately resistant (Xiao et al., 2018). Earlier studies also reported intermediate resistance to susceptibility among 16 *T. urartu* accessions (Ma et al., 1997). Some progress have also been made in elucidating the genetic basis of stripe rust resistance *in T. urartu.* Wang et al. (2020) identified and cloned a stripe rust resistant gene *YrU1* in *T. urartu* accession PI428309. *YrU1* encodes a coiled-coil nucleotide-binding site leucine-rich repeat (CC-NBS-LRR) protein containing N-terminal ankyrin-repeat and C-terminal WRKY domains, representing a unique ankyrin-repeat and WRKY integrated NLR protein. Through the RNA-seq analysis, and expression studies, A NAC transcription factor *TuNAC69* was shown to contribute to ANK- NLR-WRKY-NLR mediated stripe rust resistance (Xu et al., 2022). Zou et al. (2022b) also identified a novel LRR receptor-like kinase gene, *TuRLK1*, which is required for *YrU1* mediated immune responses, and it likely plays an important role in disease resistance to other pathogens.

#### 3.1.3 Root lesions

*Triticum urartu* has also demonstrated potential as a source of resistance against root lesion nematodes, caused by *Pratylenchus thornei*, a migratory endoparasitic nematode capable of causing yield losses of up to 65% in susceptible wheat cultivars (Sheedy et al., 2012). *P. thornei* infects and reproduces within the root cortex, resulting in root lesions, weakened root systems, and impaired uptake of water and nutrients from the soil (Mokrini et al., 2019). Screening of *T. urartu* germplasm identified resistance in five out of 21 evaluated accessions, with three accessions exhibiting greater levels of resistance than the partially resistant control genotype (Sheedy et al., 2012). Similar resistance has also been reported in *Aegilops tauschii*, the D-genome donor of hexaploid wheat (Thompson and Haak, 1997), as well as in *Ae. tauschii*–durum synthetic hexaploid wheats (Thompson, 2008).

### 3.2 Quality traits

#### 3.2.1 Endosperm storage protein

In wheat, baking properties are controlled by endosperm storage proteins grouped into glutenins and gliadins (Payne, 1987). Dough elasticity properties are determined by glutenins, while extensibility and nutritional quality are determined by gliadins (Payne et al., 1984). Glutenins are controlled by high molecular weight glutenin subunits (HMW-GSs) and low molecular weight glutenin subunits (LMW-GSs) loci (Payne, 1987). Several glutenins (Ahmadi et al., 2018; Hu et al., 2010; Luo et al., 2015; Spetsov and Daskalova, 2024) and gliadin alleles (Zhang et al., 2015) coding for endosperm storage have been identified in *T. urartu* using gene prediction, PCR-based cloning and allele specific markers. Accessions collected from the Fertile Crescent show a high diversity of HMW-GSs in *T. urartu* from Turkey compared to those from Lebanon and Syria (Caballero et al., 2009; Talini et al., 2020). Both tetraploid and hexaploid wheat have a number of known HMW-GSs and LMW-GSs loci located on group one chromosomes (Payne, 1987), however, identification of novel glutenin and gliadin alleles has the potential to further improve wheat end-use quality. Hu et al. (2010) identified a novel active gene (*FJ404595*) coding the y type HMW-GS at the *Glu-A1* locus of *T. urartu*. Expression analysis showed similar electrophoretic mobility with the y-type subunit, *1Dy12*, from the reference variety Chinese spring (Hu et al., 2010). In hexaploid wheat, the y gene in the *Glu-A1* loci is completely silent (D’Ovidio, et al., 1996; Halford et al., 1989), and hence not able to express any HMW-GSs to contribute to end-use quality. On the contrary, the gene is active in some *T. urartu* and *T. turgidum* accessions (Bai et al., 2004; Jiang et al., 2009; Waines and Payne, 1987). Bai et al. (2004) isolated a complete coding sequence of an expressed active *1Ay* gene and expression analysis produced *1Ay* proteins that were 72% identical to *IDy12* and 90% identical to *1Ay* from *T. timopheevi* (Bai et al., 2004). Recently, *T. urartu* has shown up to 18 different patterns of HMW-GS with a total of six *1Ax* alleles and eight *1Ay* alleles (Talini et al., 2020). Availability of DNA markers to efficiently detect the *1Ay* gene and distinguish the three *Glu-A1* alleles in common wheat increases the potential of utilisation of the gene for improving end-use quality (Dong et al., 2017). Further, *T. urartu* accessions have also shown a high genetic variability in the i-type (Ahmadi et al., 2018; Cuesta et al., 2017; Luo et al., 2015), m-type and s-type (Cuesta et al., 2017; Luo et al., 2015) LMW-GSs at the *Glu-A3* locus compared with the *Glu-A3* of cultivated wheat. An s-type gene, *TuA3-460*, was the first to be identified at the *Glu-A3* locus of *T. urartu* (Luo et al., 2015). A total of 11 novel alleles associated with *Glu-A3–1* genes, showing differences with those of common wheat, have been identified (Cuesta et al., 2015).

#### 3.2.2 Starch synthesis

Variability evaluation of *Wx* (waxy protein) genes of *T. urartu* and einkorn wheat (*T. monococcum* L. ssp. monococcum) accessions with the *Wx-A1a* allele of bread wheat revealed four different novel alleles (*WxA^u^1b, -A^u^1c, -A^u^1d and -A^u^1e*) in *T. urartu* accessions (Ortega et al., 2014). Waxy proteins are enzymes responsible for the accumulation of amylose during development and synthesis of starch granules in wheat (Zi et al., 2018). A full coding sequence (Guzman and Alvarez, 2012) and partial sequences (Yan and Bhave, 2000) of waxy protein genes have been reported in *T. urartu*. Recently, a novel basic zipper (Bzip) transcription factor, *TubZIP28* (*TRIUR3_00571*), on the short arm of the group 2 chromosome of *T. urartu* was reported to be expressed in the endosperm throughout grain filling. Overexpression of *TubZIP28* in wheat increases starch content by up-regulating transcription and activity of a starch synthesis related gene, cytosolic *AGPase*. Knockout of the *TubZIP28* in *T. aestivum* homologue (*TabZIP28*) using the CRISPR/Cas9 system, resulted in reduction of total starch of mature grains and reduction in thousand kernel weight (Song et al., 2020).

### 3.3 Physiological traits

#### 3.3.1 Photosynthetic traits

Photosynthetic traits are determinants of biomass production and subsequent grain yield (Richards, 2000). Wild relatives can be utilised for the improvement of photosynthetic traits because of the high degree of natural variation in key photosynthetic traits at the accession level (McAusland et al., 2020). A high rate of flag leaf photosynthesis was reported in *T. urartu* compared to its tetraploid and hexaploid relatives. Difference in flag leaf photosynthesis was attributed to ploidy level with diploid wheats having the highest rate, followed by tetraploids and then hexaploids (Austin et al., 1982). High photosynthetic rates were also identified in *T. urartu*, when photosynthesis was expressed per unit leaf area, and a much higher rate compared to hexaploid wheat when photosynthesis was expressed on a leaf dry weight basis (Austin et al., 1986). *T. urartu* also shows a high photosynthetic rate expressed in terms of high values of stomatal conductivity, high values of intercellular CO_2_ content and high values of chlorophyll content in the flag leaf (Wang et al., 2008).

#### 3.3.2 Abiotic stress resilience traits

Drought, salinity and temperature stresses are among the abiotic stresses that cause substantial losses in wheat production across the globe. Abiotic stress usually affects plant growth and development and grain yield and quality of wheat (Kamal et al., 2010). *T. urartu* and other diploid species exhibited drought tolerant traits based on physiological and chlorophyll fluorescence responses at the seedling stage. Under drought stress conditions, *T. urartu* shows high relative water content (RWC), highest values in stomatal conductance, drought stress tolerant index and decreased maximum quantum yield of PSII and maximum primary yield of photochemistry PSII (Pour-Aboughadareh et al., 2017). The dehydration responsive element (DRE)-binding proteins (DREB) which play a role in the signalling network that activates many abiotic stress-responsive genes, were also isolated and the DREB gene characterized from *T. urartu* accessions and other ancestral diploid wheats (Rustamova et al., 2022).

Climate change intensifies the frequency and severity of temperature extremes, including both heat waves and cold spells. These stresses disrupt wheat growth by impairing photosynthesis, reducing grain filling, and affecting flowering and pollination. As a result, wheat yields and quality are increasingly threatened, especially in regions already vulnerable to climatic variability (Riaz et al., 2025). Using BLASTP analysis, Gholizadeh and Mirzaghaderi (2020) identified polyamine oxidase (PAO) genes in *T. urartu* accessions along with *Ae. tauschii* and *T. aestivum*. PAO genes are involved in polyamine catabolism within the peroxisome, apoplast, and cytoplasm, and play crucial roles in plant growth, development, and responses to both biotic and abiotic stresses. Expression analysis of RNA-Seq data in *T. aestivum* revealed that several PAO genes respond significantly to heat and cold stress, while others are activated in response to infections such as powdery mildew, stripe rust, and Fusarium at various growth stages. The identified PAO genes in *T. urartu* could potentially be a source of novel genes for improvement of wheat to several other biotic and abiotic stresses.

*Triticum urartu* also offers valuable genetic resources for enhancing salinity stress tolerance in cultivated wheat. Recent studies have identified novel genes in *T. urartu* that are involved in responses to both biotic and abiotic stresses, highlighting its potential for climate-resilient breeding (Lu et al., 2018; Gao et al., 2017). A pathogenesis-related hybrid protein gene (*Pr-1-rk*) and a related pseudogene (*TuPr-1-rkP*) were recently identified in 30 and 39 *T. urartu* accessions, respectively. Transcriptional analysis revealed that *TuPr-1-rk* is expressed in response to salinity stress and pathogen attack (Lu et al., 2018). Additionally, two novel Vesicle-Inducing Protein in Plastids 1 (*VIPP1*) genes, *TuVipp1* and *TuVipp2*, were cloned from *T. urartu* accessions (Gao et al., 2017). VIPP1 proteins are known to help maintain chloroplast membrane integrity under heat (Zhang et al., 2016) and salt stress (Huang et al., 2006). In hexaploid wheat, *TuVipp1* and *TuVipp2* were found to be strongly induced under light, salt, mannitol, and cold treatments (Gao et al., 2017).

#### 3.3.3 Pre-Harvest sprouting resistance

Pre-harvest sprouting (PHS), the premature germination of physiologically mature grains in the spikes under wet conditions before harvest, is one of the major wheat production challenges affecting yield, grain and end use quality worldwide (Mares and Mrva, 2014; Tian et al., 2026). Resistance to PHS is primarily mediated through seed dormancy, a complex adaptive trait regulated by multiple genes, hormonal, and environmental factors that control the timing of germination (Finch-Savage and Leubner-Metzger, 2006; Graeber et al., 2012). In wheat, variation in PHS resistance is largely associated with differences in seed dormancy, which is linked to multiple genes and signalling pathways, particularly those involving abscisic acid (ABA) and gibberellic acid (GA) metabolism and signalling (Mares and Mrva, 2014; Rodríguez et al., 2015). Several key genes underlying PHS resistance have been cloned in wheat, including *TaPHS1* on chromosome 3AS (Nakamura et al., 2011; Liu et al., 2013), *TaMKK3-A* at the *Phs-A1* locus on chromosome 4AL (Torada et al., 2016) and recently, TaP*14K-2A* (Tai et al., 2024), and *TaCNGC-2A* on chromosome 2AL (Tian et al., 2026). These genes regulate seed dormancy through interconnected hormonal, kinase-mediated, and calcium-signalling pathways. In natural ecosystems, seed dormancy functions as an adaptive mechanism that prevents germination during transient or unfavourable environmental conditions, thereby optimizing the timing of seedling establishment and enhancing plant fitness. Wild relatives have evolved diverse dormancy strategies that allow seeds to respond to environmental cues such as temperature and moisture, ensuring germination occurs when the probability of seedling survival is greatest (Finch-Savage and Leubner-Metzger, 2006; Graeber et al., 2012; Zhang et al., 2022). Although direct studies on PHS in *T. urartu* are limited, Liu et al. (2015) showed that wild A-genome progenitors, including *T. urartu* accessions, predominantly retain the functional *TaPHS1* allele associated with stronger seed dormancy and enhanced PHS resistance, whereas domesticated wheat species possess mutations that reduce dormancy and increase susceptibility to PHS. The genetic diversity preserved in *T. urartu* may have potential to provide novel alleles that enhance resilience to PHS while contributing to wider adaptation to climatic uncertainty. However, despite the identification of functional alleles of dormancy associated genes in *T. urartu*, phenotypic evaluation of variation for pre-harvest sprouting resistance across diverse *T. urartu* germplasm remain limited. Further phenotyping and genetic studies are therefore needed to exploit this trait.

## 4 Introgression breeding and introgression of *T. urartu* into wheat

Introgression breeding involves the transfer of genetic material from one species into the gene pool of another through hybridization and repeated backcrossing (Anderson, 1953; Thórsson et al., 2001). Introgression lines are generated when homoeologous chromosomes of the two species recombine during meiosis. The polyploid nature of wheat makes it possible to introgress genetic material from its progenitors and wild relatives due to a genetic buffering effect (Dubcovsky and Dvořák, 2007; Hao et al., 2020). Studies have shown that recombination between homologous genomes of bread wheat and related species have been relatively frequent for the A and B genomes (Levy and Feldman, 2022). Two approaches can be employed in introgression breeding; a whole genome approach or transfer of targeted chromosome segments for specific regions of the genome carrying target genes (Moore, 2014). A transfer of leaf rust resistance genes from *Ae. umbellulata* into wheat through irradiation was among the earliest introgression breeding reported (Sears, 1956). Further reports on successful introgressions in wheat date back to the 1940s, 1950s and 1970s (O'Mara, 1947; Riley and Chapman, 1958; Schlegel and Korzun, 1997; Tsunewaki, 1964; Zeller and Sears, 1973) when rye (*Secale cereale* L.) segments were transferred into wheat (Rabinovich, 1997). Important traits such as high yield (Villareal et al., 1995) and resistance to powdery mildew, stem rust and leaf stripe (McIntosh et al., 2011; Rabinovich, 1997) have been associated with the 1BL.1RS translocation. By the 1980s, approximately 50% of CIMMYT wheat varieties carried the 1BL.1RS translocation, contributing to improved root biomass and broad-spectrum disease resistance (Rajaram et al., 1983). Since the successful transfer of *Ae. umbellulata* and rye segments into wheat, several other alien genome segments have been transferred into wheat. King et al. (2019) used the whole genome approach to transfer *Am. muticum* segments into wheat. The wheat/*Am. muticum* introgression lines developed have been exploited for resistance to different species of leaf rust, stem rust and yellow rust (Fellers et al., 2020) and fusarium head blight (Steed et al., 2022).

A major challenge to introgression breeding is the low level of pairing and recombination between wheat and wild relative chromosomes due to the presence of the *Ph1* (Pairing Homoeologous 1) locus located on the long arm of chromosome 5B (Okamoto, 1957: Riley and Chapman, 1958; Sears and Okamoto, 1958). *Ph1* controls the pairing of homologues to form bivalents during the process of meiosis (Riley and Chapman, 1958). In introgression breeding, *Ph1* results in univalent chromosomes at metaphase 1 (Gill et al., 1993). Switching off or deletion of the *Ph1* locus (Sears, 1977) was shown to induce homoeologous recombination between wheat chromosomes and related species (Riley and Chapman, 1958; Charpentier et al., 1988). Chinese Spring with the *Ph1* gene deleted has been successfully used in different introgression programs globally. Notably, natural suppression of *Ph1* has been reported in *Aegilops speltoides* and *Am. muticum* (Dover and Riley, 1972; Dvořák et al., 2006).

Another challenge in introgression breeding is linkage drag, which occurs when the chromosomal segment introduced from a donor parent carries not only the desired trait but also adjacent undesirable genes. These linked genes can negatively affect key agronomic traits such as yield, grain quality, and adaptability (Hospital, 2001). Linkage drag is particularly common when the donor species is genetically distant from cultivated wheat, as limited recombination between homologous chromosomes makes it difficult to separate beneficial genes from deleterious ones (Hao et al., 2020; Cuthbert et al., 2006). To mitigate linkage drag, repeated backcrossing combined with marker-assisted selection is usually employed to reduce the size of the introgressed segment and recover the elite genetic background (Hospital, 2001). Techniques such as fine mapping also allow precise localization of the target gene, enabling the selection of recombinants with minimal donor DNA (Cuthbert et al., 2006). More recently, gene editing technologies like CRISPR/Cas9 have emerged as powerful tools to directly introduce beneficial alleles into elite varieties, bypassing the need for large-scale introgression and effectively eliminating linkage drag (Dhugga, 2022).

**Figure 3:**
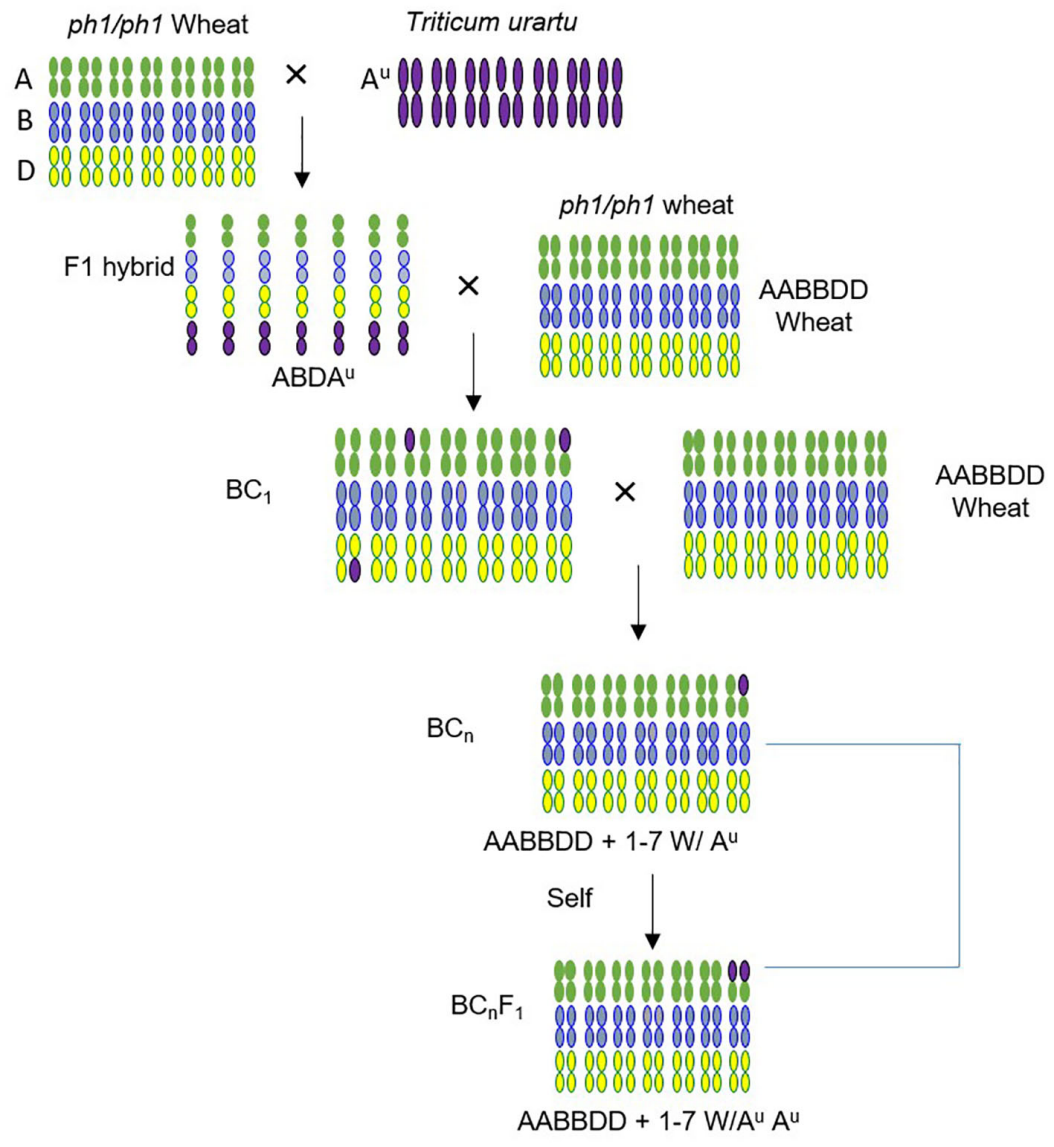
A pre-breeding crossing program for developing wheat-*T. urartu* introgression lines using the *ph1* mutant approach. W/A^u^ = heterozygous wheat/*T. urartu* recombinant, W/A^u^A^u^ = wheat/*T. urartu* homozygous recombinant.

Interspecific and intergeneric hybridisation through controlled pollination remains the primary way in which novel genes are introduced into cultivated crops (Dempewolf et al., 2017; Li et al., 2020). Despite some minor hybridisation challenges, *T. urartu* has been successfully introgressed into diploid (Fricano et al., 2014; Ma et al., 1997; Valkoun, 2001; Zhang and Batley, 2020), tetraploid (Ahmed et al., 2014; Johnson and Dhaliwal, 1976; Rafique et al., 2012; Rodriguez-Suarez et al., 2011), and hexaploid wheat (Alvarez et al., 2009; Grewal et al., 2018; Qiu et al., 2005). Direct hybridisation of *T. urartu* with hexaploid or tetraploid wheat (**Figure 3**) is possible because *T. urartu* and cultivated wheat belong to the primary gene pool of wheat, whose species can inter-mate freely to produce fertile hybrids (Chaudhary et al., 2014; Grewal et al., 2021). Furthermore, the A^u^ chromosomes of *T. urartu* are homologous to the A chromosomes of hexaploid wheat (Harlan and de Wet, 1971) maintaining synteny and macro-collinearity in majority of linkage groups with the exception of the 4A/5A/7B translocation and subsequent inversions in 4A found in the hexaploid and tetraploid wheat (Chapman et al., 1976; Devos et al., 1995). *T. urartu* genome and many other triticeae and wild relative species also have the 4A/5A translocation suggested to have originated from a common ancestor (Dvořák et al., 2018; King et al., 1994; Ling et al., 2018). However, the subsequent translocation involving chromosome 7B and inversions in chromosome 4A may make it difficult *for T. urartu* introgression from linkage group 4 to occur in the wheat background (Dvořák, 1978; Grewal et al., 2018). Although no cross incompatibility has been observed in the hybridisation of *T. urartu* and other wheats, several studies have demonstrated high levels of infertility in the F_1_ generation depending on whether *T. urartu* was used as the male or female parent. A cross between *T. monococcum and T. urartu* produced mostly sterile F_1_ plants when *T. urartu* was used as a pollen donor, while *T. urartu* as a female failed to produce viable F_2_ plants (Grewal et al., 2021). Hexaploid wheat-*T. urartu* recombinant lines generated using the wheat *ph1/ph1* approach showed a high level of infertility with low cross fertility percentage of F_1_ generation and normal fertility with every round of backcrossing (Fricano et al., 2014). Increasing the number of pollinated heads and embryo rescue have been shown to increase hybridisation success rate (Grewal et al., 2018; Valkoun, 2001).

### 4.1 *T. urartu* introgression lines with disease resistant traits

Although several screening studies have identified disease resistance traits in *T. urartu* accessions, only a few of these traits have been transferred to cultivated wheat. A powdery mildew resistance gene from *T. urartu* was successfully transferred into hexaploid wheat from a cross between *T. urartu* and Chinese spring. Expression of the gene in the hybrids showed full resistance to 15 *B. graminis* isolates. Using microsatellite markers, a powdery mildew resistance gene (*PmU)*, was mapped on the distal region of chromosome 7A^u^L (Qiu et al., 2005). Zhang et al. (2021) also used durum as a ‘bridge’ approach to transfer *Pm60* and *Pm60b* into hexaploid common wheat. Additionally, *T. urartu* introgression lines have shown resistance to different races of powdery mildew at different growth stages (Ahmed et al., 2014; Rafique et al., 2012; Valkoun, 2001; Zhang et al., 2022). For example, Amphiploids derived from crosses between *T. urartu* and durum wheat have shown varying levels of resistance to stripe rust. Ahmed et al. (2014) found that 13.4% of 194 *T. urartu* and *T. monococcum* derived amphiploids were resistant (IT 0-3) at the seedling stage, 4.63% intermediate resistance, and out of the resistant amphiploids, 8.76% of these retained resistance at the adult stage.

### 4.2 Introgression lines with quality traits

In recent years, genetic biofortification has emerged as an important breeding objective in wheat, owing to its potential to provide a sustainable solution to micronutrient. According to the World Health Organisation, approximately 2 billion people are affected by micronutrient deficiencies worldwide with the majority residing in sub-Saharan Africa (SSA) and South-east Asia (WHO, 2009; Gupta et al., 2020). At CIMMYT, synthetic hexaploid wheat derived from crossing improved tetraploid wheat (*T. durum*) or wild tetraploid *T. dicoccon* accessions with *Aegilops tauschii*, the D-genome donor of wheat has contributed to the development of elite high-zinc (Zn) wheat lines containing to 8–12 mg kg^–1^ higher grain Zn concentration above the baseline level of 25 mg kg^–1^ (Singh et al., 2017). Similarly, our preliminary screenhouse evaluation of *T. urartu* doubled haploid lines carrying different *T. urartu* segments in Paragon wheat backgrounds revealed substantial variation in grain Zn, Fe and Se under both high Zn low pH soils, and low Zn high pH soils, suggesting a potential contribution of the wild introgressed segments to micronutrient accumulation (Guwela et al., 2024a). In another preliminary study, BC_1_F_3_ *T. urartu* introgression lines in Malawian wheat backgrounds exhibited increases in grain Zn and Fe ranging from 11–30 and 8–12 mg kg ^-1^ above their recurrent parents, respectively (Guwela et al., 2024b). These findings show a high potential of *T. urartu*-derived introgression lines for enhanced grain micronutrient concentrations and their possible utilisation in wheat biofortification breeding programs. Previously, another preliminary evaluation of *T. urartu* introgression lines developed from crossing *T. urartu* with *T. monococcum* or *T. boeoticum* showed higher grain Zn content above their recurrent parents (Ma et al., 1997).

Introgression lines derived from the amphiploid between durum wheat and *T. urartu* carrying the 1Ax + Ay subunits from *T. urartu* showed higher values of gluten strength. Gluten strength was also associated with grain colour where red grains had high gluten strength while yellow grains showed soft gluten (Ma et al., 1997). The 1Ay subunit from *T. urartu* also increased glutenin content in transgenic barley lines, although it resulted in reduced gliadin content and failed dough formation due to the lack of the x-type HMW-GS and reduced number of subunits (Alvarez et al., 2009).

### 4.3 Drought and heat stress resistance lines

An introgression line derived from a cross between *T. urartu* and durum wheat showed higher yield potential and drought tolerance index compared to their recurrent parent under drought stress conditions (Fricano et al., 2014). In two season trials conducted in two different locations in Morocco and Sudan, Durum wheat lines derived from *T. urartu* showed enhanced grain yield under drought and heat stress, outperforming their recurrent parents by up to 196% (Aberkane et al., 2020). Analysis of the agronomic and yield components data of these lines showed variation in heading time and kernel weight, traits that contributed significantly to yield under stress conditions.

### 4.4 Doubled haploid production in *T. urartu*

Doubled haploids (DHs) are plants derived from cells containing a single set of chromosomes (haploid), which subsequently undergo chromosome doubling to produce a completely homozygous genome (Jacquier et al., 2020; Yel et al., 2023; Inagaki, 2003). This process enables the rapid fixation of genetic traits and is widely used in plant breeding to accelerate the development of uniform lines. DH induction can be achieved through several techniques, including anther or microspore culture, where immature pollen (microspores) are cultured to form haploid embryos, and wide hybridization, such as crossing wheat with maize or other species (Tadesse et al., 2013; Srivastava and Bain, 2018; Guan et al., 2014; Chaudhary et al., 2005). In wheat, DH plants are generated when the gametic chromosome set (n = 3x = 21) is doubled to restore the diploid state (2n = 6x = 42), resulting in complete homozygosity (Barclay, 1975; Eliby et al., 2022). In introgression breeding, DH technology is particularly valuable for generating genetic translocations, substitutions, and chromosome addition lines (Tadesse et al., 2013). It also facilitates the rapid fixation of recombined germplasm, offering a faster alternative to repeated selection of heterozygous materials, which typically requires multiple generations to achieve homozygosity at target loci (Inagaki, 2003; Jacquier et al., 2020; Tadesse et al., 2013).

Recently, we developed several doubled haploid lines with different chromosome segments of *T. urartu* for trait analysis (Grewal et al., 2018, Grewal et al., 2021). The *T. urartu* DH lines were developed by crossing *ph1* mutant hexaploid wheat with different *T. urartu* accessions. The F1 interspecific hybrids were backcrossed with the recurrent parent, and the advanced BC lines were subjected to the DH procedure. Initial studies resulted in the development of a panel of 17 wheat-*T. urartu* recombinant lines with introgressed segments covering the whole genome of *T. urartu.* Further work resulted in the generation of 86 stably inherited wheat-*T. urartu* introgression lines (Grewal et al., 2018). These lines have been phenotyped for grain micronutrients and a few promising lines have been identified (Guwela et al., 2024a, Guwela et al., 2024b).

## 5 Role of molecular markers in unlocking *T. urartu* genetic diversity for wheat improvement

The detection and characterization of *Triticum urartu* accessions and introgression lines have historically relied on a range of molecular markers. Early studies utilized microsatellite markers (Rodriguez-Suarez et al., 2011), random amplified polymorphic DNA (RAPD) markers (Qiu et al., 2005; Vierling and Nguyen, 1992), and amplified fragment length polymorphisms (AFLPs) (Chabane and Valkoun, 1998) to identify beneficial alleles, distinguish *T. urartu* from related diploid species, and assess allelic diversity. Microsatellite markers, particularly those developed for common bread wheat, demonstrated high transferability to diploid wheat species, enabling their use in *T. urartu* diversity studies (Bai et al., 2004). Wang et al. (2017) and Abbasov (2018) reported significant genetic variation within *T. urartu* using these SSR markers. SSRs have also been applied to differentiate functional and non-functional alleles at disease resistance loci, such as *Pm60a* and *Pm60a′* (Zhao et al., 2020). Further studies employing RAPDs, AFLPs, and SSRs revealed substantial genetic variability in *T. urartu* and *T. boeticum*, in contrast to the more genetically uniform *T. monococcum* (Castagna et al., 1997; Hammer et al., 2000). These markers also facilitated the distinction of *T. urartu* from other polyploid wheat species (Takumi et al., 1993; D’Ovidio et al., 1994; D’Ovidio et al., 1996), despite their shared homology and morphological similarities with the A genome. Castagna et al. (1997) used RAPD and RFLP markers to identify *T. urartu* accessions closely related to the A genome of polyploid wheat, though the presence of multiple genomes in polyploid species introduced additional bands, complicating interpretation and inflating genetic distance estimates (Castagna et al., 1997).

Despite their utility, these traditional markers did not provide comprehensive coverage enough for proper characterisation and understanding of the *T. urartu* genome (King et al., 1994). The introduction of single nucleotide polymorphism (SNP) markers marked a significant advancement in the detection of wild relative introgressions (Rodriguez-Suarez et al., 2011). The development of the Axiom^®^ Wheat-Relative SNP Genotyping Array enabled high-throughput identification of introgressions from species such *as T. urartu* (Winfield et al., 2016). Grewal et al. (2021) and Burridge et al. (2017) applied this array to hexaploid wheat backgrounds, generating a genetic map of *T. urartu* with 368 SNPs across its seven chromosomes. However, the array’s inability to distinguish heterozygous from homozygous individuals in self-fertilized populations led to the conversion of SNPs into chromosome-specific Kompetitive Allele-Specific PCR (KASP) assays (Grewal et al., 2018). These assays provided accurate genotyping and localization of introgressions and have been successfully used in doubled haploid populations and derived introgression lines (Grewal et al., 2020, Grewal et al., 2021; Guwela et al., 2024b).

## 6 Chromosome-level characterisation of *T. urartu* segments in wheat backgrounds

**Figure 4:**
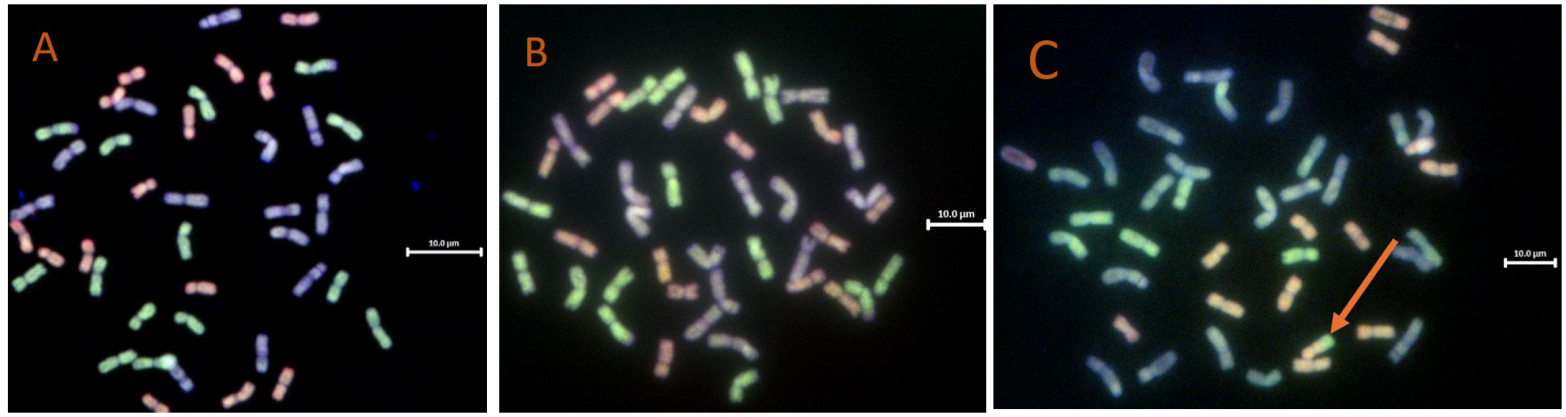
GISH from metaphase spreads showing comparisons between chromosomes of wheat and two different wheat-*T. urartu* recombinant lines in paragon wheat background. **(A)** chromosomes of hexaploid wheat. **(B)** chromosomes of an F1 hybrid derived from hexaploid wheat and wheat-*T. urartu* doubled haploid line carrying a heterozygous introgression on the A chromosome. The A chromosome cannot be distinguished from the A^u^ segments because *T. urartu* is the A genome donor. **(C)** chromosomes of an F_1_ hybrid derived from hexaploid wheat and wheat- *T. urartu* DH lines carrying a heterozygous introgression on a D chromosome. A chromosome = green, B chromosomes = blue, D chromosomes = red, A^u^ genome = Green. Source: Nottingham Wheat Research Centre.

Cytogenetic techniques such as fluorescence *in situ* hybridisation (FISH) and genomic *in situ* hybridisation (GISH) have been widely used to detect the presence of alien chromatin of different species in a wheat background (Grewal et al., 2021; He et al., 2017), and to study evolutionary chromosome rearrangements in wheat (King et al., 2019). GISH probes obtained from *T. urartu* accessions (**Figure 4**) can distinguish chromosome segments of *T. urartu* translocated to the B and D genomes of hexaploid wheat but are unable to distinguish between the A and A^u^ genomes in an A/A^u^ translocation (Grewal et al., 2018; Zhang et al., 2021).

Similarly, FISH probes showing hybridisation sites on the A genome chromosome of *T. urartu* (Grewal et al., 2021; Zhang et al., 2021) cannot clearly distinguish between all A genome chromosomes in hexaploid wheat and cannot distinguish between the A and A^u^ genomes (Adonina et al., 2015). Despite these challenges, GISH provides a visual way of counting chromosome numbers to check if introgression lines have maintained normal chromosome numbers. A combination of the cytogenetic techniques and use of molecular markers gives a better understanding of the chromosomal aberrations in the genome, following introgression hybridisation.

## 7 Conclusion and prospects

The limited genetic diversity within cultivated wheat remains a significant bottleneck in efforts to enhance wheat productivity and resilience. This constraint poses a major challenge to meeting the anticipated global food demand, which is projected to reach approximately 840 million tonnes of wheat by the year 2050, an increase from the current global production level of around 700 million tonnes. Compounding this issue are the adverse impacts of climate change and the continual emergence of novel and more virulent races of pathogens responsible for key wheat diseases, both of which threaten the stability and sustainability of wheat yields. Historically, wild relatives and ancestral progenitor species of wheat have played a pivotal role in broadening the genetic base of cultivated wheat, particularly through the introduction of resistance genes against major biotic stresses. For instance, the 2NS translocation derived from *Aegilops ventricosa* has been widely deployed in modern wheat breeding programs, conferring resistance to multiple diseases including the devastating wheat blast. Similarly, genes introgressed from wild relatives such as *Aegilops tauschii* and *Thinopyrum species* have provided valuable resistance to stem rust (e.g., Sr33, Sr45).

To meet future demands for both food quantity and nutritional quality under increasingly variable climatic conditions, it is imperative to develop wheat cultivars that exhibit enhanced adaptability to a wide range of biotic and abiotic stresses. In addition to stress tolerance and yield potential, traits related to processing quality, baking performance, and nutritional value must also be prioritized in wheat improvement programs. Encouraging progress has been made in the introgression of beneficial traits from wild species into cultivated wheat, facilitated by the availability of cost-effective and high-throughput molecular tools for tracking these introgressions. Among the promising genetic resources, *Triticum urartu* stands out as a valuable donor species for the improvement of both tetraploid and hexaploid wheat. The development of pre-breeding lines incorporating diverse *T. urartu* introgressions represents a critical step toward harnessing its genetic variability. However, further efforts are needed to phenotype these lines under a range of stress conditions, identify those with desirable agronomic traits, and conduct in-depth genetic studies to uncover the underlying genes or quantitative trait loci (QTLs) associated with these traits.

Moreover, translating this genetic diversity into elite, widely adaptable cultivars will require sustained breeding efforts. Establishing and maintaining robust pre-breeding programs that utilize progenitor and wild wheat species such as *T. urartu* necessitates substantial financial investment. Key activities requiring further investment include genome sequencing, genotyping, and advanced molecular cytogenetic analyses. Therefore, increased support from funding agencies and stakeholders is essential to ensure the continuity and success of these initiatives. Building the technical capacity of scientists in the fields of wide crossing, molecular cytogenetics, and pre-breeding is equally important. Facilitating the exchange of germplasm developed through these programs will further enhance the utilization of genetic diversity and accelerate the discovery of genes associated with important agronomic traits. Ultimately, the continued characterization and deployment of traits discussed in this review hold significant potential to contribute to the development of wheat cultivars with novel agronomic and quality attributes, thereby supporting global food and nutritional security.
